# Atypical Chronic Myeloid Leukemia: Where Are We Now?

**DOI:** 10.3390/ijms21186862

**Published:** 2020-09-18

**Authors:** Elena Crisà, Maura Nicolosi, Valentina Ferri, Chiara Favini, Gianluca Gaidano, Andrea Patriarca

**Affiliations:** Division of Hematology, Department of Translational Medicine, Università del Piemonte Orientale and Azienda Ospedaliero-Universitaria Maggiore della Carità, Via Solaroli 17, 28100 Novara, Italy; maura.nicolosi@med.uniupo.it (M.N.); valentina.ferri@uniupo.it (V.F.); chiara.favini@med.uniupo.it (C.F.); gianluca.gaidano@med.uniupo.it (G.G.); andrea.patriarca@med.uniupo.it (A.P.)

**Keywords:** Atypical CML, next generation sequencing, target therapy

## Abstract

Atypical chronic myeloid leukemia, *BCR-ABL1* negative (aCML) is a rare myelodysplastic syndrome (MDS)/myeloproliferative neoplasm (MPN) with a high rate of transformation to acute myeloid leukemia, and poor survival. Until now, the diagnosis has been based on morphological grounds only, possibly making the real frequency of the disease underestimated. Only recently, new insights in the molecular biology of MDS/MPN syndromes have deepened our knowledge of aCML, enabling us to have a better molecular profile of the disease. The knowledge gleaned from next generation sequencing has complemented morphologic and laboratory WHO criteria for myeloid neoplasms and can provide greater specificity in distinguishing aCML from alternative MDS/MPN or MPNs. The most commonly mutated genes (>20%) in aCML are *SETBP1*, *ASXL1*, *N/K-RAS*, *SRSF2*, and *TET2*, and less frequently (< 10%) *CBL*, *CSFR3*, *JAK2*, *EZH2*, and *ETNK1.* Several of these mutations affect the JAK-STAT, MAPK, and ROCK signaling pathways, which are targetable by inhibitors that are already in clinical use and may lead to a personalized treatment of aCML patients unfit for allogeneic transplant, which is currently the only curative option for fit patients. In this review, we present two emblematic clinical cases and address the new molecular findings in aCML and the available treatment options.

## 1. General Concepts

Atypical chronic myeloid leukemia (aCML) is a rare *BCR-ABL1* negative myelodysplastic syndrome (MDS)/myeloproliferative neoplasm (MPN) with a high rate of transformation to acute myeloid leukemia (AML), and is historically characterized by poor survival. The challenges of aCML management affect both diagnosis, due to the heterogeneity of the disease clinical features and the absence of unique biomarkers, and treatment choices, since no current standards of care exist.

Here we describe two representative aCML clinical cases presenting in two different age groups and review the clinicopathologic features and management of the disease. The clinical characteristics and outcome of the two patients are summarized in Table 1.

## 2. Clinical Case 1: aCML in Younger Patients

**Figure 1:**
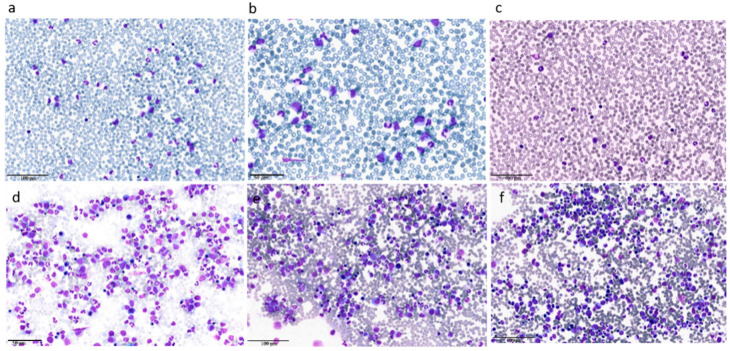
Peripheral blood (PB) and bone marrow (BM) smears of patient 1. Panels **a**-**b**-**c**: the PB shows hyperleukocytosis with neutrophil precursors (promyelocytes, myelocytes, and metamyelocytes) representing ≥ 10% of the leukocytes, with monocytes < 10% and rare blasts. The neutrophils show pseudo-Pelger-Huet nuclear abnormalities and hypogranulated cytoplasm. Panels **d**-**e**-**f**: the BM is hypercellular due to an increase of neutrophils and their precursors. The myeloid/erithroid ratio is > 10:1 due to myeloid hyperplasia. Of note, the myeloid lineage shows evident signs of dysplasia similar to those present in the PB. Moreover, the megakaryocytes show signs of dysplasia with micro-megakaryocyte and cells with hypolobated nuclei.

A 41 year old male with an unremarkable previous medical history had been diagnosed with type 2 diabetes mellitus five years before presenting to our Hematology department. He was referred to our institute due to the presence of leukocytosis. On complete blood count, white blood cells (WBC) were 41.5 × 10^9^/L with absolute neutrophil count (ANC) 34.2 × 10^9^/L, hemoglobin (Hb) was normal and platelets (Plts) were 103 × 10^9^/L. Manual differential revealed 46% hypogranulated and hypolobated neutrophils, 12% band forms, 12% metamyelocytes, 6% monocytes, 6% myelocytes, 2% promyelocytes, 2% eosinophils, and 14% lymphocytes. No hepatosplenomegaly was noted on physical examination. A bone marrow (BM) examination and trephine biopsy were performed and revealed hypercellularity with a significant myeloid expansion without excess of blasts and with mild dysplasia. Dyserythropoiesis and dysplastic megakaryocytes, including hypolobated forms, were noted (Figure 1). Karyotype was normal and a first level molecular assessment for *BCR/ABL1* rearrangement and mutations of *JAK2V617F, CARL*, and *MPL* did not reveal any genetic abnormalities.

As a next step, the diagnostic sample was subjected to next generation sequencing (NGS) analysis using TruSight Myeloid Sequencing Panel (Illumina), covering 54 genes mutated frequently in myeloid malignancies (see Supplementary Table S1), which revealed two pathogenic variants in *TET2* (p.Q635*, VAF 37.09% and p.C1221Y, VAF 42.23%) and one pathogenic mutation in *EZH2* (p.R690H, VAF 82.89%). After confirming the diagnosis of aCML, the *ETNK1* gene was sequenced by Sanger method and resulted wild type.

## 3. Clinical Case 2: aCML in the Elderly Patient/Unfit for Allogeneic Stem Cell Transplantation

A 71 year old man was referred to our Hematology department due to leukocytosis (WBC 17.7 × 10^9^/L, ANC 14.7 × 10^9^/L). He had a previous history of transitional cell carcinoma treated with radical cystectomy almost three years before referral and in complete remission ever since. In his medical history, he had a first hematology referral due to erythrocytosis at the time of the bladder neoplasia. At that time, a complete, first level molecular biology assessment for Philadelphia negative (Ph-) MPN was carried out and resulted negative, leading to the conclusion of a spurious erythrocytosis. An automated differential showed 90% neutrophils. The manual differential revealed 30% neutrophils with Pelger-Huet nuclear abnormality, 17% band forms, 6% metamyelocytes, 8% monocytes, 15% myelocytes, 14% promyelocytes, 1% blasts, and 9% lymphocytes. Fluorescence in situ hybridization (FISH) for t(9; 22) and molecular assessment for *BCR/ABL1*, *JAK2V617F*, *CARL*, and *MPL* were tested again at this time, and all resulted negative. The bone marrow (BM) aspirate was hypercellular without increased blasts but with subtle dyserythropoiesis and dysmegakaryopoiesis primarily consisting of hypolobated megakaryocytes with separate nuclear lobes (Figure 1). The BM biopsy was hypercellular (95%) with a myeloid:erythroid ratio of 5:1. Cytogenetic analysis was normal and there was no Ph chromosome, confirming the FISH result obtained on the peripheral blood (PB). NGS analysis revealed pathogenic mutations in *SETBP1* (p.D868N; VAF 47.1%), *SRSF2* (p.P95H; VAF 52.34%), and *TET2* (p.Y1245Lfs*22; VAF 52.8%). A new mutation (p.H243P) in one of the hotspots described by Gambacorti-Passerini et al. [1] was detected by Sanger sequencing and further confirmed the aCML diagnosis.

## 4. Characteristics of aCML at Presentation and Prognosis

aCML is a disease of the elderly since the median age at presentation is around 70 years old [2]. The disease is characterized by predominance in the male sex, though the biological reasons underlying aCML gender preference are poorly understood.

The clinico-pathologic characteristics of aCML include splenomegaly and a neutrophilic leukocytosis with left shift and prominent granulocytic dysplasia (e.g., hypogranular and hypolobated neutrophils, abnormal chromatin clumping, pseudo–Pelger-Huet neutrophils) [3]. The WBCcount is > 13 × 10^9^/L, with immature granulocytes > 10% of leukocytes, and < 20% blasts in the PB and in the bone marrow [4]. Due to the high WBC count, it is not uncommon to observe a monocyte count > 1 × 10^9^/L, but the percentage of monocytes is low and < 10% of the leukocytes. Basophilia is not prominent (< 2% PB basophils), and the leukocyte alkaline phosphatase level may be low, normal, or increased, therefore lacking diagnostic utility [3,5]. A hypercellular BM with myeloid hyperplasia and prominent granulocytic dysplasia is a consistent feature, multilineage dysplasia may be present [6,7].

The median overall survival (OS) of aCML patients is between 12 and 25 months [5,8,9,10,11,12]. Almost 40% of patients progress to secondary acute myeloid leukemia (sAML), with a median time to leukemic evolution of 11.2 months [10]. Unfavorable prognostic factors for OS are an increased WBC count (>50 × 10^9^/L) at presentation, increased immature precursors in the PB, female gender, and age > 65 years [2,8,10,13]. In addition, mutations in *ASXL1*, *SETBP1*, and *TET2* genes have been associated with a more aggressive disease [2,14].

The risk of progression to sAML seems to be higher in case of palpable hepato- or splenomegaly, monocytosis, BM blastosis > 5%, marked dyserythropoiesis, and transfusional requirement [10].

## 5. Diagnostic Tools in aCML

Atypical CML is a challenging myeloid malignancy with features of both myeloproliferative and myelodysplastic syndromes. The MDS/MPN category was introduced in the 2011 WHO classification to include myeloid neoplasms with clinical, laboratory, and morphologic features that overlap MDS and MPN [3,4]. In addition to aCML, this subgroup includes chronic myelomonocytic leukemia (CMML), juvenile myelomonocytic leukemia (JMML), and a provisional entity within the MDS/MPN unclassifiable group, termed as refractory anemia with ring sideroblasts and thrombocytosis (RARS-T) [4]

Currently, the diagnosis of aCML is reached primarily on morphology-based criteria, as its clinical and molecular features overlap with those of other myeloid malignancies. No molecular abnormality detected to date is specific of aCML, and multiple mutations are often present in various combinations, due to the malignant multi-step clonal evolution of myeloid malignancies [15,16].

The main feature characterizing aCML is the presence of neutrophilic leukocytosis and marked dysgranulopoiesis. Moreover, to fulfil the diagnostic criteria, WBC should be ≥ 13 × 10^9^/L with ≥ 10% of immature granulocytes, and ≤ 20% blasts in the blood and the BM [3,9].

These findings can distinguish aCML from CNL, that presents with higher WBC count (> 25 × 10^9^/L), fewer immature granulocytes (< 10%), no granulocyte dysplasia, and no excess of blasts.

Basophilia and monocytosis may be present, but, unlike Ph^+^ CML, basophils are minimally increased, and, unlike CMML, monocytes are less than 10% of the leukocytes. To rule out other myeloproliferative disorders, *BCR-ABL1* rearrangement should be excluded in all cases, whereas *PDGFRA, PDGFRB, FGFR1* rearrangements or *PCM1-JAK2* fusions must be excluded if eosinophilia is present [4] (Table 2).

Nevertheless, distinguishing aCML from other Ph- myeloid neoplasms can still be very challenging, and given its generally worse prognosis, this is a clinically important distinction, especially in transplant-eligible patients [17].

The diagnostic work up should start with complete blood count with manual differential, morphological examination of PB smear, BM examination with assessment of dysplastic features, cytogenetic analysis and FISH to exclude Ph chromosome and rearrangements involving *PDGFRA* (4q12), *PDGFRB* (5q31–33), *FGFR1* (8p11), or *JAK2* (9p24).

Once CML has been excluded, second level testing should include human leukocyte antigen (HLA) typing in transplant eligible patients, and assessment with a NGS myeloid panel, not only to confirm diagnosis but also to open the possibility of a target therapy.

**Figure 2:**
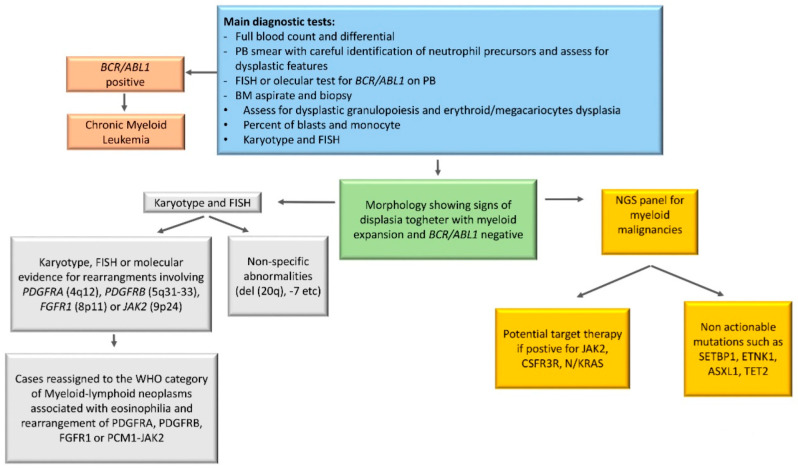
**Diagnostic algorithm for aCML.** The diagnostic work up should start with complete blood count with manual differential, morphological examination of PB smear, BM examination with assessment of dysplastic features, cytogenetic analysis, and fluorescence in situ hybridization (FISH) to exclude Ph^+^ chromosome and rearrangements involving *PDGFRA* (4q12), *PDGFRB* (5q31–33), *FGFR1* (8p11), or *JAK2* (9p24). Second level testing should include NGS with a myeloid panel, not only to confirm diagnosis but also to open the possibility of identifying druggable targets. PB, peripheral blood; BM, bone Marrow; FISH, fluorescence in situ hybridization.

An algorithm summarizing the diagnostic work-up is reported in Figure 2.

## 6. Cytogenetics in aCML

The molecular features of aCML include an increased frequency of gene fusions or an aneuploid karyotype. One or two chromosomal aberrations, e.g., trisomy 8 or 9, del (20q), −7/7q or isochromosomes 17q, are found in up to 50% of patients [2,18] but are non-specific and similar to cytogenetic findings detectable also in MDS.

Although in many myeloid malignancies there is prognostic significance associated with abnormalities of chromosome 7 and complex cytogenetics, no study to date has reported their prognostic role in aCML or in other MDS/MPN, with the exclusion of CMML.

## 7. Molecular Landscape of aCML

The heterogeneity of aCML, both clinical and molecular, is partly due to the difficulty in comparing reports of this rare disease in case series characterized over several decades, during which diagnostic criteria were redefined and novel DNA sequencing techniques emerged.

The knowledge gleaned from NGS has complemented morphologic and laboratory WHO criteria for myeloid neoplasms and can often provide greater specificity in distinguishing aCML from alternative MDS/MPN or MPNs [8]. However, a recent large study on the genetic assessment of MDS/MPN overlap syndromes has shown that CNL, aCML and CMML share, at least to a certain extent, a unique pattern of combinatorial pathway mutations that are not observed in other hematological malignancies. The overlap among genetic mutations and diseases is complex, and no single mutation or combination of mutations defines a singular disease sub-type.

The mutational landscape of MDS/MPN represents an overlap between those of MDS and MPN. Similar to MDS, the majority of cases of MDS/MPN harbors at least two mutations involving epigenetic regulation and splicing factors in the dominant clone, with acquisition of additional mutations over time [16]. Moreover, mutations in *ASXL1* and *TET2*, a frequent finding in aCML, correlate with older age and monocytosis (*TET2*), are related to “clonal hematopoiesis of indeterminate potential” (CHIP), and are present in the founder clones of both MDS and MPN. [2]. Mutation in EZH2, as shown in our clinical case 1, may also correlate with the presence of a pre-existing MDS/CHIP clone, as they are seen in only a 10% of MDS/MPN cases and never associated with *TET2* mutations [2]. The molecular architecture of CNL/aCML/unclassifiable/CMML is complex, as more than half of the patients carry co-occurring mutations in ≥ 2 different pathways involving chromatin modifiers, epigenetic regulators, splicing complex, and signaling genes [16].

**Figure 3:**
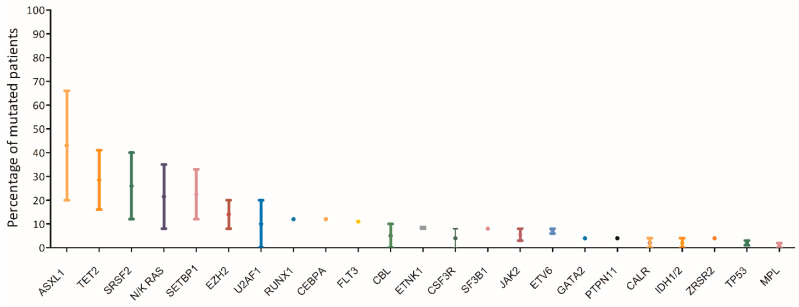
Mutational landscape of aCML. The main genes found to be mutated in aCML are represented on the X axis of the graph. Bars represent the minimum and maximum percentage of mutated patients for each gene reported in different study populations [1,2,8,18,19,20,21,22].

The most commonly affected genes (> 20%) in aCML are *SETBP1*, *ASXL1*, *N/K-RAS*, *SRSF2*, and *TET2*, and less frequently (< 10%) *CBL*, *CSFR3*, *JAK2*, *EZH2*, and *ETNK1* [1,2,8,19,20,21,22,23,24] (Figure 3). Usually the *JAK2*, *CALR*, and *MPL* genes are wild type, although a few cases of CMML and aCML have been reported to harbor *JAK2* mutations [15,25].

Mutations in *SETBP1* and *ETNK1* appear to be the alterations most closely associated with aCML, even though they are not univocally disease-specific [4,18,21,22,26]. As highlighted by the WHO-2016 revised criteria, mutations in *SETBP1* and *ETNK1* may be useful diagnostic tools for the disease [4,18,21,22,26].

Recurrent mutations of *SETBP1* have been identified in up to one third of aCML patients as compared to 10% only of MDS/MPN-U and 6–15% of CMML, and occasionally in JMML or AML secondary to MPN or MDS [27]. Multiple studies have also shown that mutations in *SETBP1* are associated with a more adverse clinical presentation (higher leukocyte count, lower Hb level, thrombocytopenia), suggesting that this alteration is not only relevant to leukemic oncogenesis, but also provides important prognostic value [20].

In 2015, recurrent mutations in *ETNK1* were identified at a prevalence of 8.8% in sixty-eight cases of aCML. All mutations were heterozygous, and in two cases occurred concomitantly with *SETBP1* mutations. ETNK1 encodes an ethanolamine kinase, catalyzing the first step of the phosphatidylethanolamine biosynthesis pathway, which is critical for regulating the cell membrane architecture and the topology of transmembrane domains of membrane binding proteins [1]. The exact functional role of *ETNK1* mutations is not completely understood. However, mutations appear to be relatively specific to aCML, as they are otherwise reported only in few cases of CMML and of systemic mastocytosis with associated eosinophilia [28].

By contrast, mutations in the *CSF3R* gene, initially described in both CNL and aCML [29], appear to be more specifically associated with CNL, in which these mutations occur in 50 to 80% of cases according to different patient series. Conversely, *CSF3R* mutations are less frequent in aCML, being found in < 10% of patients, and are rare in MDS/MPN-U [2,8,27]. Therefore, the detection of a pathogenic variant of *CSF3R* should prompt a careful revision of the diagnosis to rule out the possibility of CNL, as the molecular status would otherwise suggest.

Additionally, mutations in *SFSR2*, especially in the context of monocytosis, may orient the diagnosis towards CMML, in which this gene is mutated in up to 50% of cases.

In conclusion, NGS studies could be useful for confirming the clonality of the disease, especially in cases with mild proliferative features and concomitant inflammatory comorbidities that might mimic a reactive leukocytosis process, and may help in the differential diagnosis among different MDS/MPN categories together with morphologic and clinic data.

**Figure 4:**
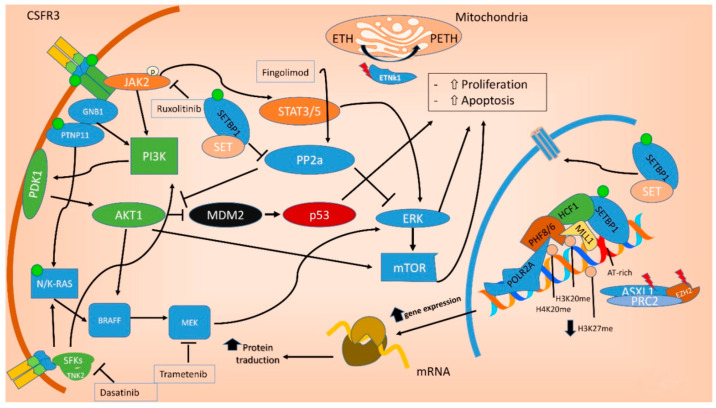
Molecular pathways involved in aCML. Mutations in the *ASXL1* and *EZH2* genes are mainly point mutations that lead to impaired function of the polycomb repressive complex 2 (PRC2), which translate in decreased epigenetic repression of key genes involved in stem cell renewal, thus promoting myeloid proliferation and differentiation. Mutations of both *NRAS* and *PTNP11* result in a constitutive activation of MAPK, promoting cancer cell survival and proliferation. *SETBP1* encodes a protein named SET binding protein 1 (SEB) that regulates the SET inhibitory activity on tumor suppressors, including PP2A. In aCML, all the *SETBP1* mutations result in an increased gene expression and, through SET, in a reduction of PP2A inhibitory activity on AKT and MAPK pathways, leading to increased cellular proliferation and survival. Fingolimod targets PP2A with an activating effect. *ETNK1* encodes an ethanolamine kinase, which catalyzes the first step of the de novo phosphatidylethanolamine biosynthesis pathway, critical for regulating membrane architecture and the topology of transmembrane domains of membrane binding proteins. Due to the fact that the ethanolamine kinase 1 contributes to different processes in the cell, the mechanisms by which the mutant protein induces myeloproliferation have not yet been clarified. *CSFR3* mutations may be membrane proximal mutations or truncation mutations or a combination of the two. All the activating missense mutations target the proximal domain leading to increased dimerization and activation of JAK-STAT pathway, sensitive to its kinase inhibitor ruxolitinib. Conversely, *CSFR3* truncating mutations induce receptor signaling through SRC family kinase rendering the cells sensitive to the multikinase inhibitor dasatinib. Green dot: activating mutation; red lightning: inactivating mutations. ETH: Ethanolamine; PETH: phosphatidylethanolamine.

Moreover, the molecular findings may have crucial relevance in directing personalized therapies, since aCML-associated genetic alterations might be susceptible to specific therapeutic approaches, directly targeting the mutant proteins or their associated pathways (Figure 4).

Indeed in the last decade, the identification of oncogenic mutations in signal transduction proteins has clarified that specific pathways regulating the proliferation of myeloid lineage cells may be deregulated in leukemia [27]. Several mutations commonly found in aCML impact on the JAK-STAT, MAPK, and ROCK signaling pathways, that are known to be responsible for myeloproliferation in physiological conditions and to be aberrantly activated in myeloproliferative diseases [30], including Acml [9,27] (Figure 4). For example, *NRAS*, *JAK2*, and *CSF3R* mutations directly impact on the MAPK and JAK/STAT pathways; *SETBP1* encodes a protein named SET binding protein 1 (SEB) that impacts on AKT and MAPK pathways; mutations in the *PTPN11* gene result in constitutively activated RAS signaling [30]. Inhibitors targeting signal transduction components of the pathways mentioned above are already in clinical use and have the potential to be used for personalized treatment of aCML patients.

In addition, in myeloid disorders, and particularly in MDS, mutations in several genes are adverse prognostic factors, namely alterations of *ASXL1*, *RUNX1*, *EZH2*, and *TP53* [31,32,33]. It is likely that mutations in these genes might also emerge as adverse prognostic factors in MDS/MPN.

Finally, the identification of molecular markers may be useful to monitor minimal residual disease (MRD), as validated in other myeloid diseases and shown in a few studies of aCML [34,35].

## 8. Clinical Case 1: Treatment Choices in a Transplant Eligible Patient

**Figure 5:**
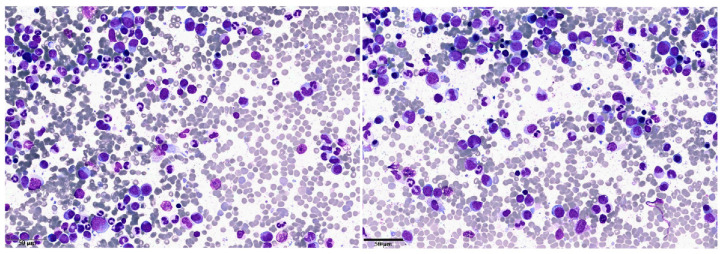
Bone marrow smears after 3 cycles of decitabine of clinical case 1. The reduction of marrow blasts with persistence of trilineage dysplasia and a residual thrombocytopenia are consistent with an optimal marrow response according to proposed international criteria [36].

Due to progressive leukocytosis and monocytosis, a BM assessment was repeated, documenting a stable disease without increase of blasts. However, considering the young age of the patient, the progressive leukocytosis despite cytoreduction and the progressive monocytosis that has been correlated with a higher risk of progression to AML [10,23], we opted for decitabine therapy (20 mg/m^2^ IV for 5 days on 28 day cycles) as an off-label prescription. After 3 cycles, the patient achieved a partial hematological response [36] with a normalization of WBC count but a residual thrombocytopenia. A BM aspirate showed no excess of blasts with persistent trilineage dysplasia (Figure 5). Because no matched related donors were available, a matched unrelated donor (MUD) search was opened. A 10/10 unrelated donor was identified, and the patient proceeded to a myeloablative hematopoietic stem cell transplant (HSCT) conditioned with busulfan and cyclofosfamide. The patient remains in a hematologic and molecular remission at 3 months after transplant with mild chronic graft-versus-host disease GVHD.

## 9. Clinical Case 2: Treatment Approach in a Transplant Ineligible Patient

The patient was considered unfit for HSCT due to age and to the previous diagnosis of solid neoplasia. Although the patient’s WBC count was only mildly elevated and the Hb and Plts count were well preserved, his long term prognosis was poor due to the general high risk of AML progression related to the diagnosis of aCML itself and the unfavorable prognosis associated to his molecular profile, specifically with *SETBP1* mutations and the generally poor risk related to *SRSF2* alterations in the context of other myeloid neoplasms. Unfortunately, all the mutations detected in this case were not actionable, therefore pegylated interferon alpha (Peg–IFNα) 3 mcg/kg/week was chosen as first line therapy. After 3 months of treatment, a partial response was achieved and maintained over time with no toxicity.

## 10. Treatment

**Figure 6:**
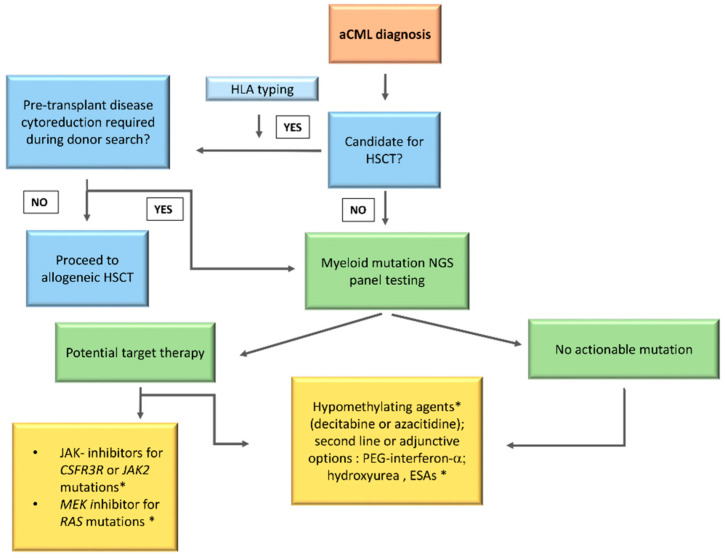
Treatment algorithm for aCML. This flow-chart summarizes the treatment management of aCML. Treatment choices are made according to few key variables: eligibility for allogeneic hematopoietic stem cell transplantation (HSCT); mutations identified by next generation sequencing (NGS) panel; availability and eligibility for clinical trials; possibility to adopt therapies used for myelodysplastic syndrome (MDS) or MPN (e.g., hypomethylating agents or second-line/adjunctive therapies). * Patients should be enrolled in a clinical trial whenever possible.

No standard of care is currently available for aCML management. This rare entity of MDS/MPN, as already detailed, is characterized by heterogeneous clinical and genetic features with a very poor prognosis. In addition, due to the low disease incidence and the absence of large randomized clinical trials, the physician’s choices remain challenging. Several treatment strategies validated in other myeloid diseases have been evaluated in aCML, starting from erythropoiesis stimulating agents (ESAs) to improve the anemia and up to cytoreductive drugs to control the proliferation and reduce the blast count (AML-like chemotherapy, hypomethylating agents (HMA), hydroxyurea (HU), or PEG–IFN–α. Given the recent clarification of the mutational landscape of MDS/MPN, different target therapies, including *JAK2* inhibitors, are currently being investigated. However, up to date, the only curative treatment remains HSCT, an option available for the younger patients only. Participation on clinical trials should be considered in all cases. A possible treatment algorithm is reported in Figure 6.

### 10.1. Hematopoietic Stem Cell Transplant

HSCT is considered as the sole potentially curative strategy for aCML. Data on this approach are scant and most evidence originates from studies including patients with heterogeneous MDS/MPNs. Lim et al. reported a series of 10 patients with MDS/MPN who received HSCT. The 5-year OS and disease-free survival (DFS) rates of the whole cohort were 42.2% and 46%, respectively. Only 2 out of 10 patients were aCML, and, at the median follow-up of 47.5 months, both were alive and in remission [37]. Similar OS and DFS rates (2 years OS and DFS 47% and 37%, respectively) were reported by Mittal et al. in a patient series of 20 MPD/MPN cases, including 8 aCML, who received HSCT [38]. Koldehoff et al. analyzed 9 aCML patients, receiving either sibling allogeneic donor type (*n* = 4) or MUD (*n* = 4) or a syngeneic transplantation from a twin sibling (*n* = 1) [39]. All patients were in complete remission at the median follow-up of 55 months and only one patient relapsed 19 months after syngenic HSCT but re-obtained a remission after a second allotransplant [39]. A subsequent follow-up study including 21 aCML transplanted patients reported a 5 year OS rate of 80% with a median survival of 48 months [40]. These promising results with HSCT were confirmed in a larger series of 42 aCML cases reported by Onida et al. who received HLA-identical sibling (64%) or MUD (36%) cells [41]. At a median follow-up of 89 months, 87% of patients were in complete remission with a 5 year OS rate of 51% and a relapse free survival rate of 36%.

No evidence-based information on the best timing of transplantation is currently available, and indications rely on expert opinions. Gotlib et al. proposed HSCT for all candidate patients at the time of diagnosis [19], whereas the Moffitt group suggested to stratify patients according to the known clinical (age > 65, leukocytosis > 50 × 109/L) and molecular (*SETBP1* mutation) prognostic factors and to offer HSCT at diagnosis in high risk cases and defer the procedures in the others [42].

The choice between these two approaches should also consider the availability of a suitable donor at the time of diagnosis and the presence of a targetable mutation that might be appropriate for an attempt with molecular therapies.

In conclusion, allogeneic HSCT may be considered a promising approach for a subset of aCML patients and the new molecular findings may pave the way to a MRD driven approach to treat patients after HSCT; however, a large number of cases are elderly and may be not candidates for the HSCT.

### 10.2. Hypomethylating Agents

Based on the use of the HMA azacytidine or decitabine in CMML, different studies have tried to use these drugs also in aCML [43]. Kantarjan et al. analyzed the role of decitabine in a large series of 130 patients with *BCR/ABL1* positive and negative CML. Out of seven aCML patients, four obtained a clinically meaningful response but their median survival was only 13 months and the 2 year survival rate was 14% [44]. More recently, single case reports have documented a response to decitabine in a CML patients, that in 2 cases successfully allowed a bridge to transplant [45,46,47,48]. Data on azacitidine are even more limited and less promising. Patnaik et al. reported the outcome of 4 patients who were treated with azacytidine and achieved a stable disease as best response. No patients underwent HSCT in this small series.

HMA cannot be considered as a standard of care for aCML and their use is off-label. However, these drugs represent an alternative to standard chemotherapy for the pre-transplant cytoreduction in case of a low blast count, as in our clinical case 1, or in the presence of comorbidities. Moreover, given the evidence that the use of HMA as a bridge to HSCT in MDS does not worsen the transplant outcome and OS after transplant, it is tempting to extrapolate this hypothesis also to aCML [49,50,51]. However, it must be emphasized that the response to this agent is transitory, so even if a CR is reached the transplant must not be delayed.

HMA may also be considered for patients who are ineligible to transplant that do not carry actionable somatic mutations and are intolerant to HU as an alternative to IFN.

### 10.3. AML-Like Chemotherapy

The role of AML-like intensive chemotherapy has not been explored extensively, but is usually offered to selected patients with high-risk proliferative clinical behavior as a bridge to HSCT [42].

### 10.4. Interferon-Alpha and Hydroxyurea

HU is commonly used to control splenomegaly and leukocytosis. Older studies reported that HU and IFN-alfa are able to induce a complete hematological response, however with short duration [5,11]. PEG–IFN–α–2b is a pegylated IFN with a significant advantage over non-pegylated form in the administration schedule (once a week) and toxicity profile. In a study on 38 patients with BCR–ABL–negative MPDs, which included 5 aCML, 45% of patients achieved a complete or partial response, with a median duration of response of 20 months [52]. Both HU and IFN are useful in a palliative setting, where an allogenic stem-cell transplant is not considered due to age or comorbidities. In these clinical contexts, HU and IFN may help control myeloproliferation, reduce splenomegaly and possibly, in the case of IFN, control symptoms (as in our case 2). The pegilated form of IFN should be preferred whenever possible for the patient’s convenience.

### 10.5. Target Therapy

Several promising targeted therapies are currently being investigated, including the JAK inhibitor ruxolitinib, the SRC kinase inhibitor dasatinib, and the MEK inhibitor trametinib (the main molecular targetable pathways in aCML are shown in Figure 4).

*JAK2 V617F* and *CSF3R T618I* mutated cases, although infrequent among aCML patients, might benefit from the *JAK2* inhibitor ruxolitinib, that is already in clinical use for the treatment of myelofibrosis and polycythemia vera. Preclinical studies have shown that membrane proximal mutation of *CSF3R*, like T618I, induce a lethal MPN similar to aCML and CNL in murine models, and that the administration of ruxolitinib is effective in reducing WBC, decreasing spleen weight and increasing body weight in mice [53,54]. Based on these promising results, ruxolitinib has been tested as a single agent in a few case reports with encouraging results [55,56]. Recently, in a phase II study on a series of 35 MDS/MPN, including 4 aCML, the combination of ruxolitinib and azacitidine was well tolerated and led to 57% of responses, particularly in *JAK2* mutated patients. A benefit in survival, more evident in MDS/MPN–U than in aCML, was observed. Of note, no *CSF3R* mutated patients were included in this study [57]. The results of ruxolitinib in continuum or as a bridge to allotransplant in primary myelofibrosis may further suggest its use in aCML [58,59], however, due to the limited data currently available for aCML, this agent should be recommended in the setting of clinical trials only.

In addition to activation of the JAK-STAT pathway induced by missense mutations, *CSF3R* truncating mutations activate the SRC family-TNK2 kinase signaling and are sensitive to dasatinib, as shown by in vitro studies on cell lines [29] (Figure 4). In the presence of a truncating mutation of *CSF3R*, a trial of a SRC kinase inhibitor such as dasatinib might be a reasonable approach, even if no in vivo reports have yet established its efficacy.

Another option for *NRAS/KRAS* mutated patients, accounting for one third of aCML, is trametenib, a MEK1/2 inhibitor. Use of trametinib specifically resulted in prolonged survival in mice transplanted with primary *NRAS* mutated AMLs compared to untreated mice [60]. These preclinical results were confirmed in a phase I/II study in which trametinib showed clinical activity in patients with *RAS*-mutated myeloid malignancies (AML, MDS, and CMML), with manageable or reversible toxicities [61]. A single case report describes an aCML patient harboring an *NRAS* mutation who obtained a near complete hematologic response within several months of treatment with trametinib [62]. Moreover, MEK/ERK signaling confers oncogene dependence in CSF3R induced leukemia. This suggests that targeting MEK or ERK might be effective also in *CSF3R* mutant aCML [63].

Finally, there might be a future role for activators of PPA2, a major protein phosphatase which acts as a tumor suppressor, in the setting of aCML with SETBP1 overexpression. In fact, overexpression of SETBP1 protects SET from protease cleavage, increasing the formation of a SETBP1-SET-PP2A complexes that result in PP2A inhibition, promoting proliferation of the leukemic cells. Pharmacological activation of PP2A seems to offer a future therapeutic alternative, since in vitro PP2A restoration by PADs (PP2A-activating drugs, such as fingolimod [64]) reverses some of the leukemogenic features [65].

Clinical trials including aCML patients are needed to come to clear treatment recommendation, even if the rarity of disease is an obstacle to the design of studies with high numerosity.

At the moment of manuscript preparation, only 2 trials are actively recruiting aCML patients. Both are focused on the blastic phase of aCML. One is a phase I/II trial on a combination of azacitidine, venetoclax, and pevonedistat in patients with newly diagnosed AML secondary to other myeloid diseases including aCML (NCT03862157). The second study is a phase II trial testing the combination of topotecan hydrochloride and carboplatin with or without veliparib in AML secondary to myeloproliferative disorders or aCML in advanced phase (NCT03289910).

## 11. Conclusions

The diagnosis of aCML is always a difficult task to accomplish. Until now, the diagnosis has been based on morphological grounds only, possibly underestimating the true frequency of the disease. Only recently, new insights in the molecular biology of MDS/MPN syndromes have deepened our knowledge in aCML. However, all the mutations described in aCML so far are neither specific nor diagnostic, being present in a wide range of other hematological malignancies and also in the normal elderly population, even though at a very low VAF. With a more comprehensive molecular profiling, the diagnostic criteria will be hopefully refined in the future to include also genetic features resulting in decreased reliance on absolute cutoffs in blood counts that may vary along with the disease history of the patients. Nonetheless, as discussed earlier, in some cases these alterations can be more than a simple diagnostic test and may allow personalized treatment. Large collaborative efforts are needed to design studies that will provide data so that the management of these diseases will be evidence-driven.

## Figures and Tables

**Table 1:** Characteristics at Diagnosis and Outcome of the Two Clinical Cases of aCML.

	Clinical Case 1	Clinical Case 2
Age (years)	41	71
WBC	32.12 × 10^9^/L	29.35 × 10^9^/L
Differential (PB)	hypogranulated neutrophils 46% band-cells 12% eosinophils 2% basophils - monocytes 6% lymphocytes 14% myeloblasts - promyelocytes 2% metamyelocytes 12% myelocites 6%	neutrophils with P-H abnormality 30% band-cells 17% eosinophils - basophils - monocytes 15% lymphocytes 9% myeloblasts 1% promyelocytes 14% metamyelocytes 6% myelocites 8%
Hb	13.5 g/dL	14.9 g/dL
Plts	103 × 10^9^/L	239 × 10^9^/L
BM blasts	2%	4%
Screening for mutations in *JAK2, CALR* and *MPL*	negative	negative
*BCR/ABL*	negative	negative
Gene mutations identified by NGS panel	*TET2* p.Q635* (VAF 37.09%) *TET2* p.C1221Y (VAF 42.23%) *EZH2* p.R690H (VAF 82.89%)	*SETBP1* p.D868N (VAF 47.1%) *SRSF2* p.P95H (VAF 52.34%) *TET2* p.Y1245Lfs*22 (VAF 52.8%)
Screening for *ETNK1* mutations	negative	*ETNK1* p.H243P
Karyotype	46, XY (20)	46, XY (20)
Treatment	Decitabine 3cycles PR with residual thrombocytopenia HSCT	Peghilated IFN alpha PR
Follow-up time (months after diagnosis)	8 months	10 months
Status at last follow up	Alive in complete remission	Alive with stable disease and partial hematological response

WBC, white blood cells; Hb, hemoglobin; PLTS, platelets; PB, peripheral blood; BM, bone marrow; NGS, next generation sequencing; CR, complete response; PR, partial response.

**Table 2:** WHO 2016 Diagnostic Criteria for atypical chronic myeloid leukemia (aCML) [4].

WHO 2016 Diagnostic Criteria for aCML
Peripheral blood leukocytosis (WBC count ≥ 13 × 10^9^/L) because of increased numbers of neutrophils and their precursors with prominent dysgranulopoiesis
Neutrophil precursors (promyelocytes, myelocytes, metamyelocytes) ≥ 10% of leukocytes
No Ph chromosome or *BCR-ABL1* fusion gene and not meeting criteria for PV, ET, or PMF *
No evidence of *PDGFRA*, *PDGFRB*, *FGFR1* rearrangement, or *PCM1-JAK2*
Minimal absolute basophilia; basophils usually < 2% of leukocytes
No or minimal absolute monocytosis; monocytes usually < 10% of leukocytes
Hypercellular bone marrow with granulocytic proliferation and granulocytic dysplasia, with or without dysplasia in the erythroid and megakaryocytic lineages
Less than 20% blasts in the blood and bone marrow

ET, essential thrombocythemia; Ph, Philadelphia; PMF, primary myelofibrosis; PV, polycythemia vera; WBC, white blood cell. * Cases of myeloproliferative neoplasms (MPN), particularly those in accelerated phase and/or in postpolycythemic or postessential thrombocythemic myelofibrosis, if neutrophilic, may simulate aCML. A previous history of MPN, the presence of MPN features in the bone marrow and/or MPN-associated mutations (in *JAK2*, *CALR*, or *MPL*) tend to exclude a diagnosis of aCML. Conversely, a diagnosis of aCML is supported by the presence of *SETBP1* and/or *ETNK1* mutations. The presence of a *CSF3R* mutation is uncommon in aCML and, if detected, should prompt a careful morphologic review to exclude an alternative diagnosis of chronic neutrophilic leukemia or other myeloid neoplasm.

## Supplementary Materials

The following are available online at https://www.mdpi.com/1422-0067/21/18/6862/s1, Table S1.
